# Supplementary material for: An automated pipeline for the discovery of conspiracy and conspiracy theory narrative frameworks: Bridgegate, Pizzagate and storytelling on the web
Source: PLoS One. 2020 Jun 16;15(6):e0233879. doi: 10.1371/journal.pone.0233879 (PMC7297331; doi:10.1371/journal.pone.0233879)
Supplement: S1 File — We also provide details of the results that were obtained for the two data sets studied in this paper. (PDF) [file pone.0233879.s001.pdf]

Methodology, Pipeline, and Results: Details

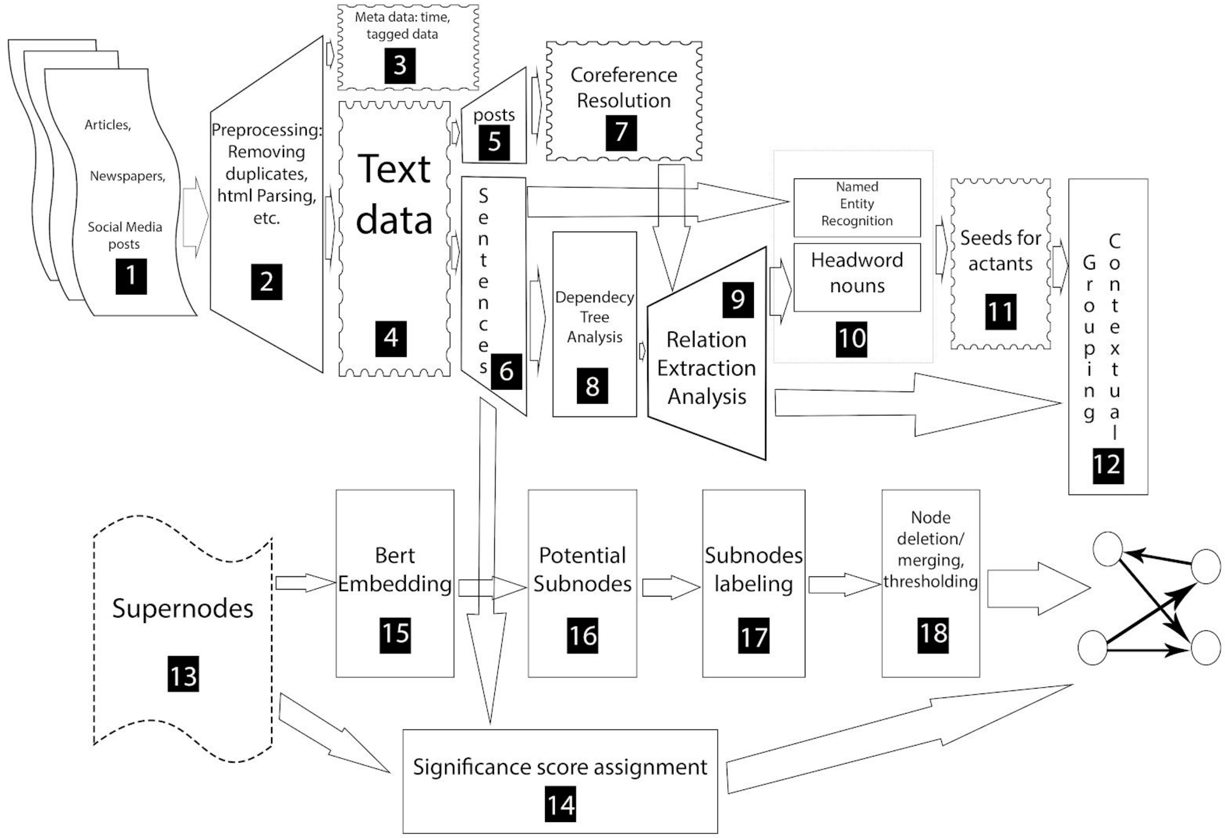

Figure 1: A flowchart of the computational steps executed in our end-to-end pipeline. The salient computational steps are described in detail below and in less detail in the methodology section of the paper.

Code for this project and instructions for running the code are available through a github repository. Access can be requested by contacting the corresponding author. https://github.com/Roychowdhury-group/FENESTRA-Fake-News-Structure-and-Threat-Assessment

Each block mentioned in Figure 1 diagram is described below:

Block 1: Data is generated from various sources, including key statistics for the two datasets

Bridgegate:

Number of documents: 385

Number of sentences: 20433

Number of dependency tree based relationships: 21667

Number of srl-based (A0,V,A1) relationships: 25693 (58% of the total SRL extractions)

Number of SRL extractions in total: 44212

Pizzagate:

Number of posts: 17,948

Number of sentences: 42979 (29790 of them has a length of greater than 30)

Block 2: Preprocessing step. We use the resources from the previous block in order to obtain noiseless, relevant and informative raw text data as pipeline input.

Tools:

warctools
pandas
boilerpipe
guess_language
adblockparser
Beautifulsoup

Some examples of the preprocessing steps include:

a. warcindex to find all HTTP responses
b. warcpayload to extract pages possibly containing useful texts
c. pandas to remove duplicate URLs
d. removal of spam web pages

Block 3: One of the outputs from the previous step are date-time stamps. In the analysis of conspiracies and conspiracy theories, it is helpful to know the timing of events. The plot below shows how timing information can be used for tracking the emergence of different actants and events associated with them.

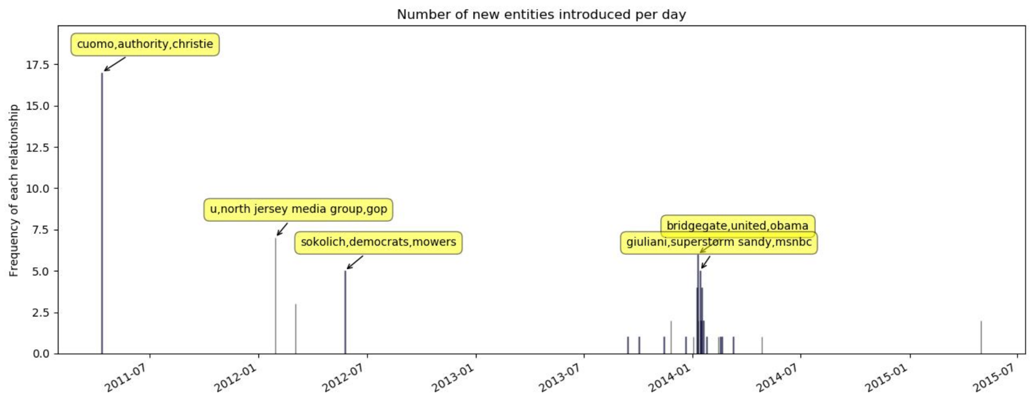

Blocks 4,5,6: Two types of output are produced in this step. First, we apply paragraph-based coreference resolution where each post is treated as a paragraph. Second, we parse each sentence individually to extract the relations, using nltk to segment paragraphs into sentences.

Block 7: Using the coref part of Stanford corenlp package, the paragraphs/posts are used as input with each input to the coref resolver comprising several sentences. The number of sentences varies given the disparate lengths of posts. The output of coreference is translated into a mapping dictionary, where each word (usually a pronoun) in a sentence is mapped to another word (ideally a non-pronoun noun). We index the corpus so that each word in paragraph has a unique sentence number and unique word ID, often corresponding to its sentence index. The mapping dictionary, as a result, is from word ID to word ID. An example of the coreference output is shown below:

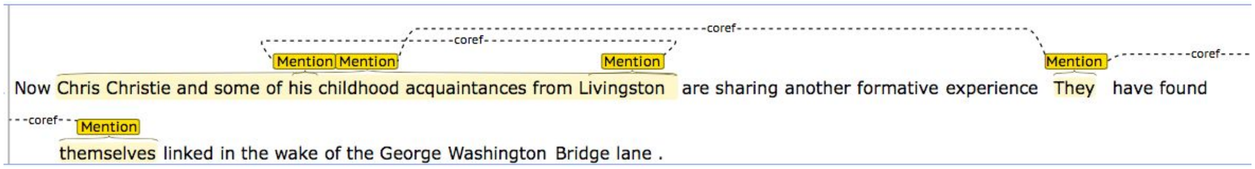

Block 8: We used the sentences from the previous step and extracted the dependency trees using the stanford corenlp package.

Block 9: In this step, we use the output of the dependency tree parsing for syntactic extractions. The detailed output is described below:

Relationship types - descriptions & examples

Our relation extraction combines dependency tree parsing and Semantic Role Labeling (SRL). We first design a set of patterns to mine relationship patterns from dependency trees. The patterns are extensions of two prior works: Ollie and ClauseIE. Second, we form extractions from SENNA's Semantic Role Labeling (SRL) model. We combine dependency-based extraction techniques together with SRL to increase the recall of our system. Then we apply cleaning and deduplication techniques to select unique and high precision extractions.

The following table summarizes our relationship extraction patterns:

Patterns	Patterns Type	Example	Derived Extraction
(nsubj, verb, dobj)	SVO	Christie fired Kelly	(Christie, Fired, Kelly)
(nsubj, verb (no obj), prep)	SVP	Wildstein resigned on Dec. 6th	(Wildstein, resigned, on Dec 6th)
(nsubj, verb, noun-cop)	SVCop	The lane closures were retribution	(The lane closures, were, retribution)
(nsubj, verb (with obj),	SV(O)P	Christie fired Kelly on Jan 8th	(Christie, fired kelly on, Jan 8th)

prep)		The lanes were shut down for a traffic study	(The lanes, were shut down, for a traffic study)
(nsubj, verb)*	SV	Biden died of heart attack	(Biden, died)
(word, appos, word)	Appos	Christie fired that aide, Bridget Anne Kelly, a deputy chief of staff.	(Bridget Anne Kelly, is, a deputy chief of staff)
(A0, Verb, A1)<br>(A0, Verb, A2)<br>(A1, Verb, A2)	SRL	Ring was uncovered by the leaked Podesta emails dumped by Wikileaks	(by wikileaks, dumped, the leaked podesta emails)<br>(by the leaked Podesta emails dumped by wikileaks, uncovered, ring)

*We take SV extraction only if subject does not come with an object or a complement, and the verb is among a set of predefined intransitive verbs such as die, or walk.

In addition to the above patterns, we have extended patterns such as (nsubjpass, verb, dobj), (xsubj, verb, dobj) by which we extract relationships from passive sentences. Also we extract relationships when there is a “conjunction and” is present. For example, from a sentence “Prosecutors have charged Kelly and Baroni.”, not only do we extract (Prosecutors, have charged, Kelly) as a SVO extraction, but also we retrieve (Prosecutors, have charged, Baroni) since the object (Kelly) is connected to another noun (Baroni) via a “conj_and” edge in the dependency tree.

Additional example sentences:

For Bridgegate:

1. Christie fired Kelly from her job in January after emails came to light connecting her to the closures.
2. Wildstein resigned on Dec. 6, calling the bridge scandal a distraction.
3. His administration initially claimed the lanes were shut down for a traffic study.
4. Bridget Kelly, the governor's former deputy chief of staff, told jurors in federal court in Newark that she discussed the plan to shut down access lanes at the George Washington Bridge with Christie.
5. The report places blame on Kelly, the deputy chief of staff Christie fired, and David Wildstein, whom Christie appointed to a post at the Port Authority.
6. Christie fired that aide, Bridget Anne Kelly, a deputy chief of staff.
7. Prosecutors have charged Kelly and former Port Authority of New York and New Jersey executive Bill Baroni, Christie's highest ranking political appointee at the transportation agency, with creating massive traffic gridlock in Fort Lee, New Jersey, as payback after the town's Democratic mayor, Mark Sokolich, refused to back Christie's 2013 reelection campaign.

For Pizzagate:

1. It claimed that Hillary Clinton and her campaign chief were running a child trafficking ring in the restaurants back rooms.
2. ring that was uncovered by the leaked Podesta Emails dumped by Wikileaks.
3. Biden died of heart attack, so did breitbart.
4. Democrats have alleged the lane closures were punishment directed at Sokolich for failing to endorse Christie in his reelection bid last year.
5. Democrats allege the lane closures were retribution against the mayor for failing to endorse Christie.

Example 1:
Sentence:
Christie fired Kelly on Jan. 8 after emails obtained by The Record showed she apparently ordered the lane closures.

Covers: SVO, SV(O)P, SRL

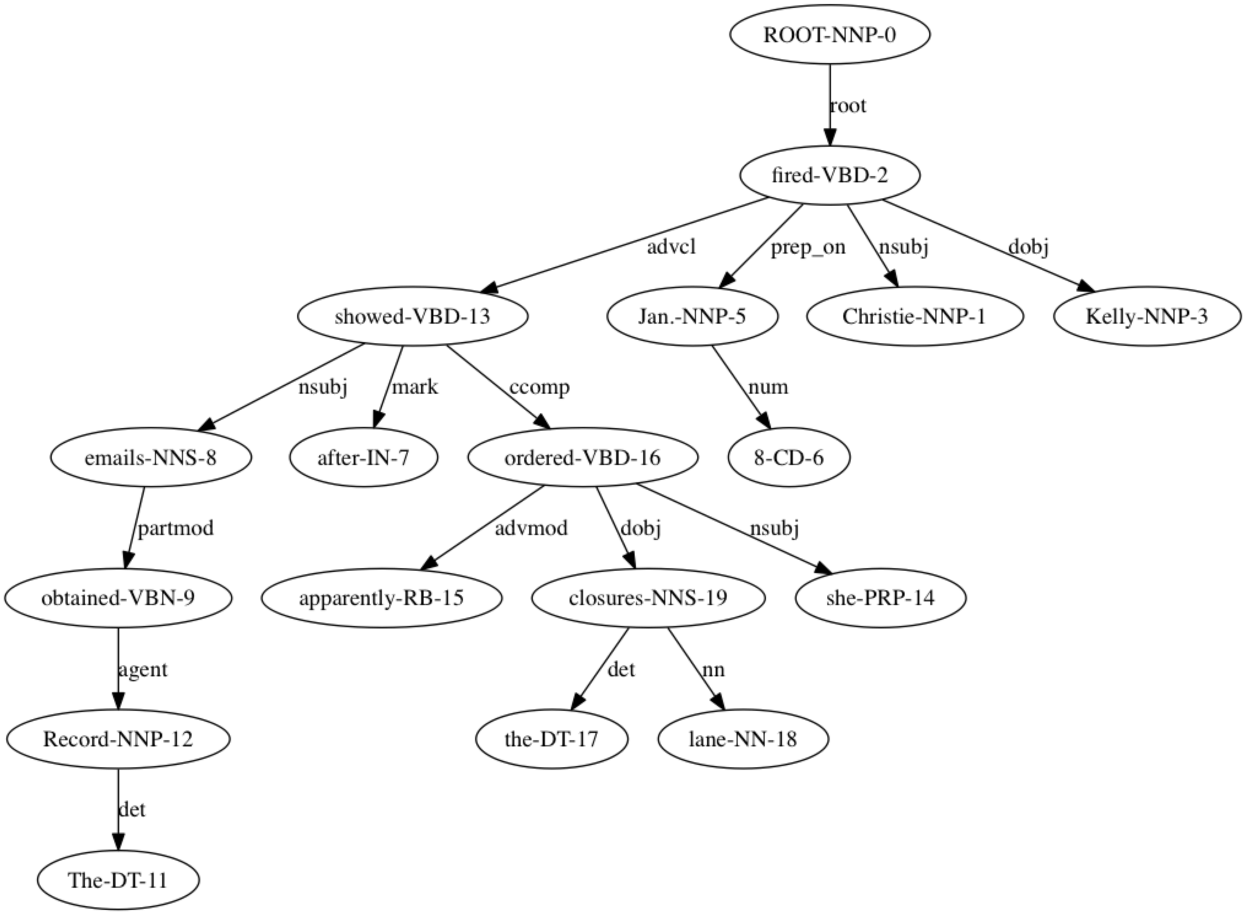

Type	Arg1	Relation	Arg2
SVO	{Christie}	{fired}	{Kelly}
	{she}	Apparently {ordered}	The lane {closures}
SV(O)P	{Christie}	{fired} <<{Kelly}>> on	{Jan.} 8
SRL	{Christie}	{fired}	{Kelly}
	By The {Record}	{obtained}	{emails}
	{emails} obtained by The Record	{showed}	{she} apparently ordered the lane closures
	{she}	{ordered}	The lane {closures}

The entire set of results from running relation extraction can be found at:
Bridgegate - Link to the complete excel file:
https://drive.google.com/open?id=1ncrtvIf5yZkEks3y0xsf4wwPtJiVN9Yc
Pizzagate - Link to the complete excel file:
https://drive.google.com/open?id=1o8RU6dgwt08oq7szO4F6X5T5-ORZmoFq

Block 10: In this step we apply Named Entity Recognition (NER) tools to find various types of named entities along with their frequencies. In addition to NER, we used headword occurrences to find mentions of nouns as concepts in our corpus. The headwords are usually the words in arguments that happen to be at a higher level in the dependency tree. In SRL, we have the relations without a dependency tree. In order to tackle this problem, we find the corresponding word IDs in the parse tree and then pick the top node in the SRL argument to use as headwords for extractions. After combining the headwords and mentions, we have almost enough seeds to cover all the arguments in our relationship graph. Examples of NER types and the aggregated final list is shown below:

	NER Type	Definition	Example
1	PERSON	People, including fictional	Christie
2	ORG	Companies, agencies, institutions	Port Authority
3	GPE	Countries, cities or states	Fort Lee
4	FAC	Facilities such as buildings, airports, bridges	George Washington Bridge
5	NORP	Nationalities or religious or political groups	Republican party

6	LOC	Non GPE locations such as bodies of water	Hudson River
7	EVENT	Named storms, wars, sports events	Hurricane Sandy
8	OTHER(ARG)	Miscellaneous entities	attorneys

Block 11: In this step, we combine the headword and NER lists to get the most important and frequent mentions. It is interesting to note that the number of mentions of entities has a power law distribution for both Bridgegate and Pizzagate. More precisely, the log-log plot of frequency vs rank is a straight line, following Zipf's law.

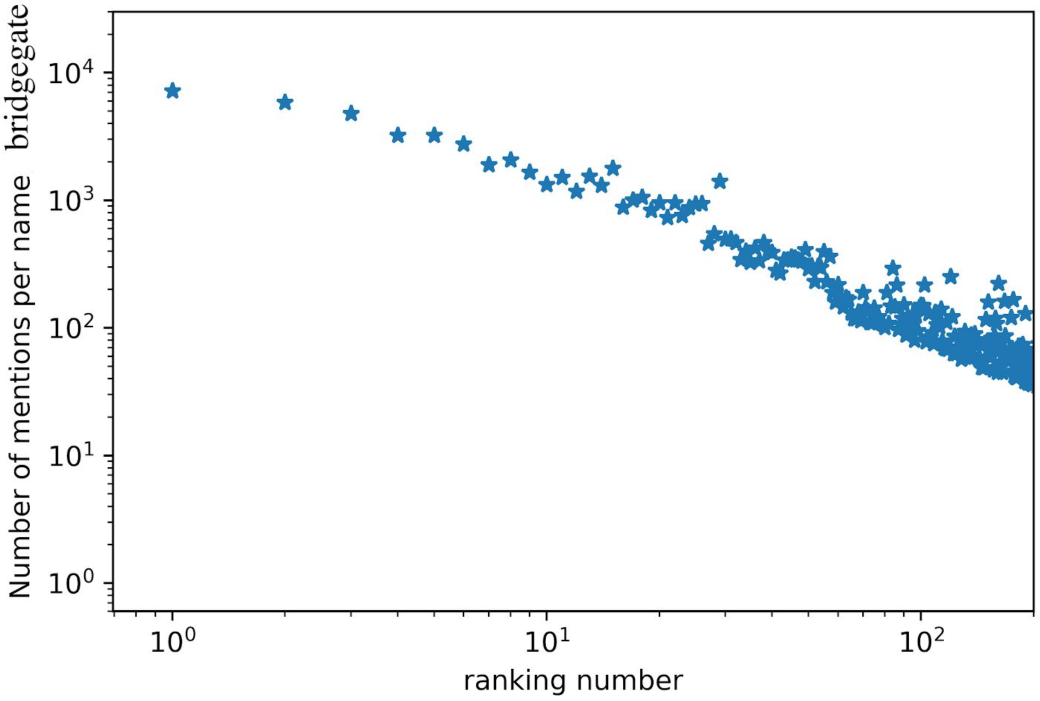

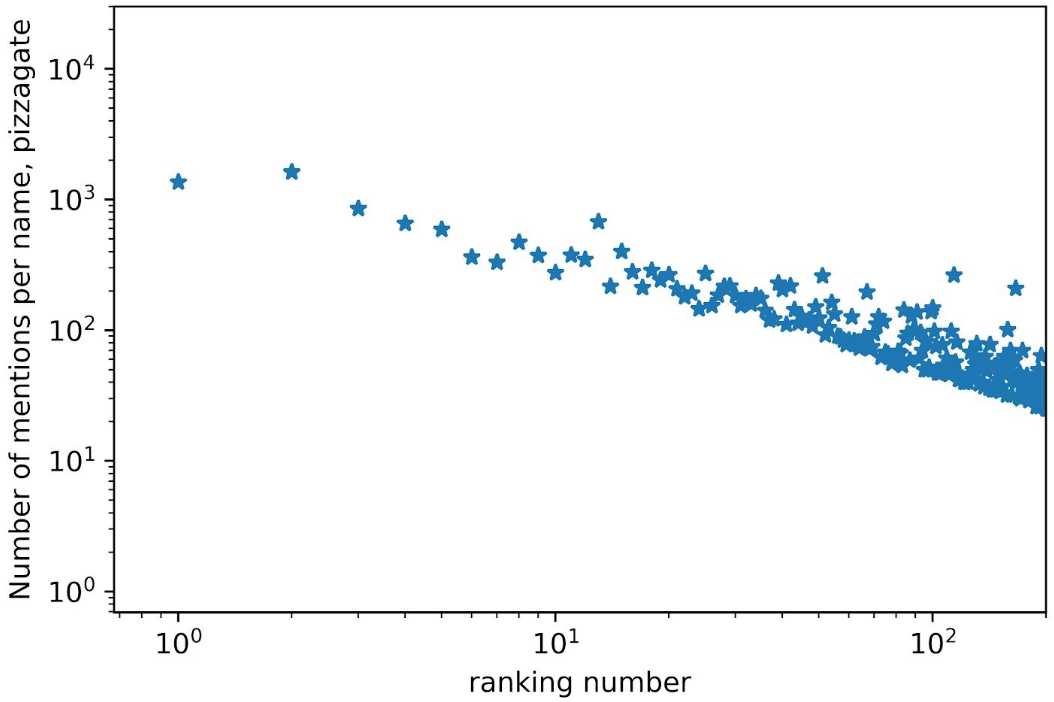

The top 100 aggregated mentions are printed below for each dataset:

Bridgegate - top 100

rank	entity	type	frequency_score_sum_NER_arg
1	christie	PERSON	23925
2	port authority	ORG	7366
3	wildstein	PERSON	5785
4	fort lee	GPE	4729
5	kelly	PERSON	4284
6	baroni	PERSON	3818
7	new jersey	GPE	3338
8	stepien	PERSON	2718
9	sokolich	PERSON	2496

10	george washington bridge	FAC	2109
11	samson	PERSON	2040
12	chris christie	PERSON	1694
13	record	ORG	1638
14	republican	NORP	1419
15	democrats	NORP	1411
16	democratic	NORP	1402
17	david wildstein	PERSON	1375
18	mastro	PERSON	1328
19	gwb	PERSON	1299
20	bridget anne kelly	PERSON	1135
21	wisniewski	PERSON	1071
22	zimmer	PERSON	1042
23	bill baroni	PERSON	958
24	governor	OTHER(ARG)	893
25	new york	GPE	880
26	bridgegate	PERSON	874
27	u	ORG	851
28	people	OTHER(ARG)	834
29	fishman	PERSON	779
30	foye	PERSON	741
31	closures	OTHER(ARG)	738
32	mark sokolich	PERSON	735
33	democrat	NORP	735
34	authority	ORG	692
35	critchley	PERSON	685
36	hoboken	GPE	676
37	report	ORG	676

38	office	ORG	672
39	republicans	NORP	663
40	documents	OTHER(ARG)	651
41	trump	PERSON	638
42	north jersey media group	ORG	628
43	weinberg	PERSON	600
44	newark	GPE	582
45	prosecutors	OTHER(ARG)	544
46	committee	ORG	540
47	drewniak	PERSON	537
48	gibson dunn	PERSON	532
49	time	ORG	523
50	bill stepien	PERSON	519
51	officials	OTHER(ARG)	514
52	scandal	OTHER(ARG)	500
53	john wisniewski	PERSON	459
54	sandy	PERSON	454
55	david samson	PERSON	447
56	email	OTHER(ARG)	447
57	gop	ORG	447
58	senate	ORG	444
59	u attorney office	ORG	440
60	giuliani	PERSON	435
61	mayor	OTHER(ARG)	431
62	investigation	OTHER(ARG)	430
63	michaels	PERSON	426
64	trenton	GPE	426
65	state	ORG	417

66	attorney	OTHER(ARG)	412
67	nixon	PERSON	409
68	agency	OTHER(ARG)	404
69	n j	GPE	404
70	firm	OTHER(ARG)	403
71	renna	PERSON	393
72	odowd	PERSON	375
73	administration	ORG	371
74	superstorm sandy	EVENT	371
75	loretta weinberg	PERSON	343
76	bridget kelly	PERSON	333
77	obama	PERSON	333
78	chief	OTHER(ARG)	330
79	legislature	ORG	323
80	randy mastro	PERSON	320
81	assembly	ORG	320
82	wigenton	PERSON	313
83	questions	OTHER(ARG)	311
84	bridge	FAC	305
85	emails	OTHER(ARG)	303
86	barbara buono	PERSON	300
87	guadagno	PERSON	298
88	baldassare	PERSON	298
89	lanes	OTHER(ARG)	295
90	gibson	PERSON	292
91	nj transit	ORG	290
92	attorneys	OTHER(ARG)	290
93	teaneck	PERSON	283

94	campaign	OTHER(ARG)	273
95	information	OTHER(ARG)	273
96	fifth amendment	LAW	272
97	dawn zimmer	PERSON	269
98	jersey	GPE	267
99	mowers	PERSON	266
100	director	OTHER(ARG)	263

Pizzagate - top 100:

rank	entity	type	frequency_score_sum_NER_arg
1	people	OTHER(ARG)	2374
2	children	OTHER(ARG)	1247
3	alefantis	PERSON	890
4	clinton	PERSON	885
5	reddit	ORG	864
6	podesta	PERSON	854
7	fbi	ORG	843
8	trump	PERSON	754
9	pizzagate	PERSON	646
10	someone	OTHER(ARG)	601
11	anyone	OTHER(ARG)	596
12	evidence	OTHER(ARG)	566
13	police	ORG	551
14	vatican	FAC	523
15	facebook	ORG	484
16	anything	OTHER(ARG)	454
17	haiti	GPE	443
18	wikileaks	ORG	441

19	things	OTHER(ARG)	432
20	information	OTHER(ARG)	425
21	jews	NORP	421
22	hillary	PERSON	416
23	something	OTHER(ARG)	413
24	washington	GPE	405
25	man	OTHER(ARG)	403
26	wikipedia	ORG	395
27	cia	ORG	384
28	catholic	NORP	378
29	voat	ORG	371
30	time	ORG	369
31	use	OTHER(ARG)	368
32	post	ORG	366
33	comet pizza	ORG	360
34	church	ORG	359
35	ring	OTHER(ARG)	357
36	alig	PERSON	351
37	story	OTHER(ARG)	347
38	america	GPE	343
39	account	OTHER(ARG)	341
40	services	OTHER(ARG)	336
41	israel	GPE	332
42	way	OTHER(ARG)	331
43	american	NORP	327
44	subreddit	OTHER(ARG)	325
45	u	GPE	323
46	united states	GPE	322

47	twitter	ORG	316
48	thing	OTHER(ARG)	313
49	guy	PERSON	309
50	msm	ORG	308
51	person	OTHER(ARG)	308
52	lot	OTHER(ARG)	308
53	hollywood	GPE	301
54	comet	ORG	295
55	pizza	ORG	293
56	james alefantis	PERSON	291
57	news	OTHER(ARG)	286
58	years	OTHER(ARG)	282
59	obama	PERSON	282
60	us	GPE	281
61	users	OTHER(ARG)	273
62	nothing	OTHER(ARG)	272
63	podestas	PERSON	271
64	investigation	OTHER(ARG)	269
65	case	OTHER(ARG)	267
66	comet ping pong	ORG	260
67	place	OTHER(ARG)	258
68	shit	OTHER(ARG)	257
69	kids	OTHER(ARG)	253
70	finders	ORG	252
71	abuse	OTHER(ARG)	248
72	site	OTHER(ARG)	245
73	trademarks	OTHER(ARG)	242
74	fbianon	ORG	239

75	jewish	NORP	237
76	dc	GPE	236
77	article	OTHER(ARG)	234
78	sra	ORG	231
79	guys	OTHER(ARG)	230
80	pedophiles	OTHER(ARG)	230
81	aquino	PERSON	229
82	victims	OTHER(ARG)	229
83	clinton foundation	ORG	225
84	everyone	OTHER(ARG)	223
85	content	OTHER(ARG)	222
86	americans	NORP	222
87	world	ORG	219
88	link	OTHER(ARG)	218
89	cp	ORG	218
90	media matters	ORG	215
91	c	GPE	215
92	clintons	PERSON	214
93	nsfwe	ORG	212
94	child	OTHER(ARG)	206
95	nyt	ORG	205
96	republican	NORP	205
97	owner	OTHER(ARG)	205
98	posts	ORG	203
99	everything	OTHER(ARG)	201
100	california	GPE	201

Blocks 12, 13: A large fraction of mentions of entities co-occur with each other. For example, ‘hillary’ and ‘clinton’ repeat in a high number of arguments. As explained in the main paper, grouping the most co-occurrent mentions in the same context helps to form initial nodes with

higher resolution. For example, if we group ‘hillary’ and ‘clinton’ in the same contextual group, then, ‘Clinton foundation’ and ‘hillary foundation’ will be in the same subnodes. We call this group of words in the same context a *supernode*. The goal of this process is to create supernodes which carry the most relevant arguments. For example ‘pizza owner’ and ‘comet owner’ are assumed to be in the same subnode at a certain granularity, since comet pizza is a frequent term in the pizzagate corpus. Therefore, if we contextually group these terms in the same supernode, we place them in the same subnode. For this purpose, we used a maximum number for limiting the number of mentions in each supernode. Here, we used 4 as the maximum number of entities to be contextually grouped to form the seed nodes in any supernode. We removed stopwords after the formation of supernodes. The results of this step are shown in step 13.

The supernode seed list for bridgegate:
nodeID nodeLabel
0 authority port executive
1 christie governor chris former
2 wildstein david
3 kelly bridget anne
4 new jersey york
5 lee fort mayor sokolich
6 baroni bill
7 democrat democrats democratic
8 stepien
9 bridges bridge george washington lane
10 samson
11 recordings records
12 bills billing
13 office officer
14 governors
15 mastro randy attorney
16 wisniewski john assemblyman dean
17 attorneys
18 michael michaels critchley
19 gwb
20 state states house weinberg loretta
21 zimmer
22 ann vardeman pollster monmouth
23 closures closure
24 committees legislative panel state
25 reporters reporting reports mastro
26 foye
27 dunn gibson crutcher lawyer

28 groups group
29 fishman paul
30 sandy superstorm aid recovery
31 investigators investigation investigations investigating investigator investigate investigative committee
32 mark marks
33 bridgegate trial
34 people
35 lanes
36 media medium group
37 emails email kelly
38 drewniak michael
39 trumped trump donald
40 document related authority
41 prosecutors federal
42 scandal scandals bridge
43 cities city
44 official officials
45 newark
46 senator senate senators
47 firms firm law samson
48 housing houses department
49 giuliani rudy
50 county counties bergen judge court
51 agencies agency
52 nixon richard new
53 united unit airline
54 lawyers
55 buono barbara democratic
56 parties party tea republican
57 laws
58 campaign campaigns
59 clinton hillary
60 obama barack president senate
61 guadagno kim constable
62 transit transition nj lease
63 chief staff
64 cuomo andrew
65 mowers matt photographer
66 duhaime
67 egea regina
68 controversy controversial controversies closure
69 statements statement

70 legislation legislator legislators
71 pat foye
72 schar reid counsel general
73 messages message text
74 transportation
75 teaneck
76 inform informant informal information
77 execution executives
78 communities community identify affair american
79 traffic study jam lee
80 police policed
81 dawn zimmer
82 hayes hay melissa
83 cases case criminal charge defense
84 commissioned commission report
85 river hudson vale south
86 issue issued issues subpoena
87 calatrava design santiago architect
88 manhattan
89 employee employees
90 developer project estate real
91 roberts robert durando onetime supreme
92 wolff
93 middlesex democrat
94 politically political politics
95 requests request comment
96 post huffington
97 voters voter
98 appointee appointees
99 rechler scott walker vice
100 marty kaplan carpenter 2014
101 closed close closings closing
102 journal journalism professor joe
103 watergate scandal
104 jams jammed
105 hampshire
106 lawmaker lawmakers
107 problems problem traffic
108 planned plan planning plans
109 patrick director
110 commissioner commissioners
111 endorsed endorsing endorse endorsements endorsement
112 tunnels tunnel lincoln

113 allege allegations alleged allegation
114 testimony testimonial baroni
115 client clients firm
116 managers management manager manage campaign
117 rutgers
118 company companies private
119 republican republicans
120 hoboken
121 mayors
122 renna christina genovese
123 gop
124 legislatures legislature
125 baldassare
126 sweeney stephen
127 marino kevin
128 affairs affair intergovernmental
129 fulop
130 academy jose marti freshman
131 msnbc
132 presidency
133 bush jeb
134 candid candidate candidates presidential
135 boburg shawn
136 wigenton susan
137 unions union
138 rockefeller
139 judges
140 verizon
141 lesniak raymond
142 cortes
143 garten

Supernode Seed lists for for pizzagate:
nodeID nodeLabel
0 podesta
1 pizza comet ping pong
2 parties party
3 clinton hillary
4 ring pedo
5 child
6 pizzagate
7 trump
8 barack obama

9 wiki WL
10 email
11 art
12 handkerchief
13 Alefantis
14 Traffick
15 haiti
16 twitter
17 instagram facebook
18 tunnel
19 satan
20 vatican
21 painting
22 monica peterson
23 scalia

Block 14: In this step, we used the method described in the main paper to 'measure' the importance of a verb occurring between two target actants. For each pair of two supernodes, we used the dependency tree to find the verbs which occur in sentences that include any mention of each supernode seed word. As explained in more detail in the paper, we assign a significance score to each verb given two supernodes. Therefore, the output is a memory based dictionary where, with the key of two target supernodes, we get a score for each verb.

Block 15: As part of the process of generating the automated graph, we collect all arguments which have at least one mention corresponding to each of the supernodes. This provides an initial form of the graph, where supernodes constitute the nodes and the corresponding relations constitute the edges. This interim graph is very noisy. For example, 'hillary clinton' and 'hillary campaign' are mapped to the same node. In order to solve this problem, we use embeddings that encode the semantics of phrases. With the help of an unsupervised k-means algorithm, we broke down the supernodes into smaller sized and more dense nodes, which we call subnodes. With this approach, we can also tune the number of clusters.

Block 16: Given the output of the k-means clustering, we have the set of subnodes.

Block 17: In order to find the best words describing each subnode, we use a scoring similar to TFIDF, where the documents are the paragraphs and the term frequencies are based on the subnode arguments. Given this score, we choose up to five top scored words.

Block 18: After finding the best word or words to describe each subnode, we merge the subnodes based on similar labels. In order to reduce the number of subnodes, we apply a thresholding based on the relative size of subnodes to the average size of nodes for every supernode. Shown below are plots for an example supernode from each corpus, pizzagate and bridgegate. In the log-log plot, the threshold is sharp:

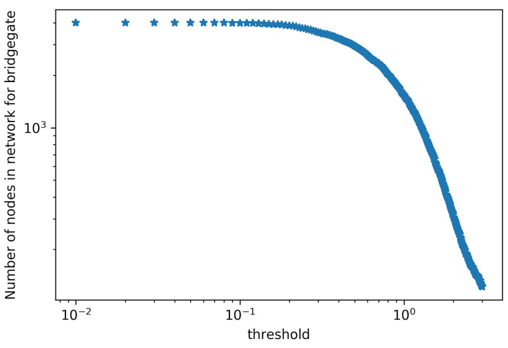

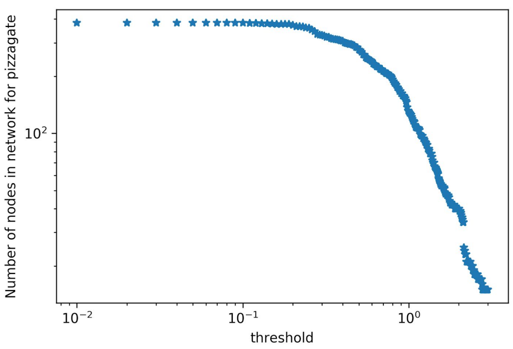

Subnode list (as defined by their automated labels) for bridgegate:

nodeID nodeLabel
0 authority port
1 Chris christie
2 Samson
3 chairman samson
4 former
5 David Wildstein
6 christie painting
7 Bill Stepien
8 Joe Peyronnin
9 Bridget Anne Kelly
10 New Jersey
11 New York
12 Mark Sokolich
13 Fort Lee Mayor
14 Bill Baroni
15 Lane Closure gwb
16 gwb Bridge Lane
17 Lane
18 Wolff Samson
19 file record

20 Attorney
21 Lee Cortes
22 committee legislative
23 Kelly's Email
24 Donald Trump
25 Bridge Scandal
26 agency
27 Philip Kwon
28 traffic jam
29 public
30 commissioner
31 hofstra peyronnin
32 hampshire
33 authority transportation
34 wisniewski
35 Rockefeller Group
36 livingston thomas
37 mastro
38 Patrick J. Foye
39 sandy superstorm
40 New Jersey
41 authority report
42 authority executive
43 Dawn Zimmer
44 News
45 Republican
46 evidence
47 reporter
48 Rudy Giuliani
49 Mike DuHaime
50 Mitsu Yasukawa
51 painting
52 Stephan Sweeney
53 Andrew M. Cuomo
54 Steven M. Fulop
55 White House
56 People
57 Document
58 GOP
59 legislature
60 counsel general
61 voter
62 director executive

63 Renna
64 Melissa Hayes
65 boburg shawn
66 Paul Fishman
67 denoia edmund
68 barbara challenger
69 Kim Guadagno
70 Susan Wigenton
71 attorney
72 action
73 Michael Critchley
74 Defence
75 bureau melissa
76 sponsored transparent
77 federal judge
78 democrat
79 monmouth murray
80 dunn gibson
81 record
82 email
83 republican
84 campaign
85 press
86 news
87 gwb
88 meeting
89 affair
90 Michael Drewniak
91 firm
92 endorsing
93 trial
94 David
95 Cortes
96 footing taxpayer
97 Paul Nunziato
98 police union
99 staff
100 trenton
101 michael chertoff
102 committee investigation
103 senate
104 police
105 story

106 Stephen Sweeney
107 state weinberg
108 chris peyronnin
109 hoboken
110 lawyer
111 Clinton
112 president
113 Regina Egea

Subnode list for pizzagate:
nodeID nodeLabel
0 tony podesta
1 pizzagate pizza
2 party
3 article part party art
4 martin stewart party
5 podesta
6 podesta podestas
7 pizzagate
8 alefantis
9 clinton
10 pedophile pedophilia
11 pedo pedos
12 pedo ring
13 child
14 trafficker trafficking child blackmaildead human
15 trafficking
16 tunnel
17 wikileaks email wiki wikipedia podesta manager
18 wikipedia wikileaks pedophile
19 email
20 email podesta leaked
21 twitter
22 satan
23 part party art article artist partner
24 instagram
25 comet story pizza
26 comet
28 pizza pizzagate
29 pedophile
30 obama
31 email single
32 article artist party participant art partner

33 handkerchief
34 satanic
35 part heart art
36 clinton contribution foundation
37 ring hearing
38 wikileaks
39 satanism satanist satanic
40 jimmycomet comet james alefantis
41 pizza
42 wikipedia
43 james alefantis
44 hillary clinton
45 part
46 party labour
47 child porn
48 clinton foundation
49 pizza comet
50 john podesta
51 hillary
52 instagram photo
53 facebook
54 pong ping comet
55 trump
56 podesta john clinton campaign hillary chairman
59 james alefantis ,instagram ,owner ,account ,comet ,
60 satanic ,ritual ,
61 cannibal ,openly ,cp ,posting ,sick ,pedo ,
62 alefantiss ,
63 trafficking ,human ,sex ,slave ,
64 pedophilia ,pedophile ,
65 satanism ,
66 tunnel ,underground ,sewage ,
67 donald ,trump ,
68 party ,casa ,podesta ,chicago ,mahubabani ,pizza ,
69 podestas ,
70 obamas ,advance ,team ,personal ,president ,
71 wl ,
72 rothschild ,child ,childrenkanye ,childporn ,16 ,12 ,
73 hovered ,satan ,smoke ,vatican ,
74 party ,labour ,child ,birthday ,councillor ,photo ,
75 trump ,epstein ,hosted ,attended ,party ,employee ,
76 ring ,
77 hunting ,trip ,scalia ,

78 podesta ,sexist ,resembling ,mccain ,abduction ,brother ,
79 party ,ingram ,thurston ,leader ,county ,paul ,
80 brother ,podesta ,
81 trafficking ,child ,rapetrafficking ,abusetrafficking ,attitude ,brotheltrafficking ,
82 party ,cooky ,3rd ,
83 owner ,alefantis ,james ,pong ,ping ,comet ,
84 haiti ,
85 cannibalism ,mutilation ,torture ,picture ,
86 twittergate ,
87 painting ,
88 cannibalism ,

The Choice of Thresholds in determining Core and Overlapping Nodes in Community Partitioning of Narrative Networks:

As described in the main paper, (i) We first perform community detection T_{max} times for a given narrative network, (ii) Then, we create a community co-occurrence matrix $A(i,j)$ = k, if nodes i and j co-occur k times in T_{max} runs, (iii) Finally, we normalize it by T_{max} to get the probability that a pair of nodes (i,j) co-occur in any random run. *If each run returned the exact same community structure*, then the normalized matrix will have only 0 or 1 values, forming an adjacency matrix. The resulting graph defined by such an adjacency matrix will be a set of cliques, where each clique represents the nodes belonging to the same community. However, due to randomness in the community partitioning algorithm, loosely connected nodes often change community assignments, and hence the normalized co-occurrence matrix has values $0 \leq A(i,j) \leq 1$. Next we use a threshold value P_{th} to create an adjacency matrix as follows: If $A(i,j) < P_{th}$ then we set it 0, otherwise if $A(i,j) \geq P_{th}$ then it is set to 1. Next we find the connected components in this graph and record the size of the Giant Connected Component (GCC) and the number of connected components. The thresholding procedure could create disconnected and isolated nodes, and we disregard such isolated nodes, as described in Algorithm 1 in the main section.

The following plot shows the case for the Pizzagate narrative network (T_{max} = 1000), recording both the GCC size (the left Y-axis), and the number of connected components (the right Y-axis) as a function of P_{th} . As one can observe, when P_{th} is set to 1, the GCC size is minimum, and as it is decreased to 0 it becomes the entire network. In between, it increases in steps, as connected components get merged. Similarly, as the threshold is decreased the number of connected components decreases until it becomes a single connected component. As one can

see from the plot, the connected components remain quite separated until around P_{th} =0.45 when many of them merge to create a large connected component.

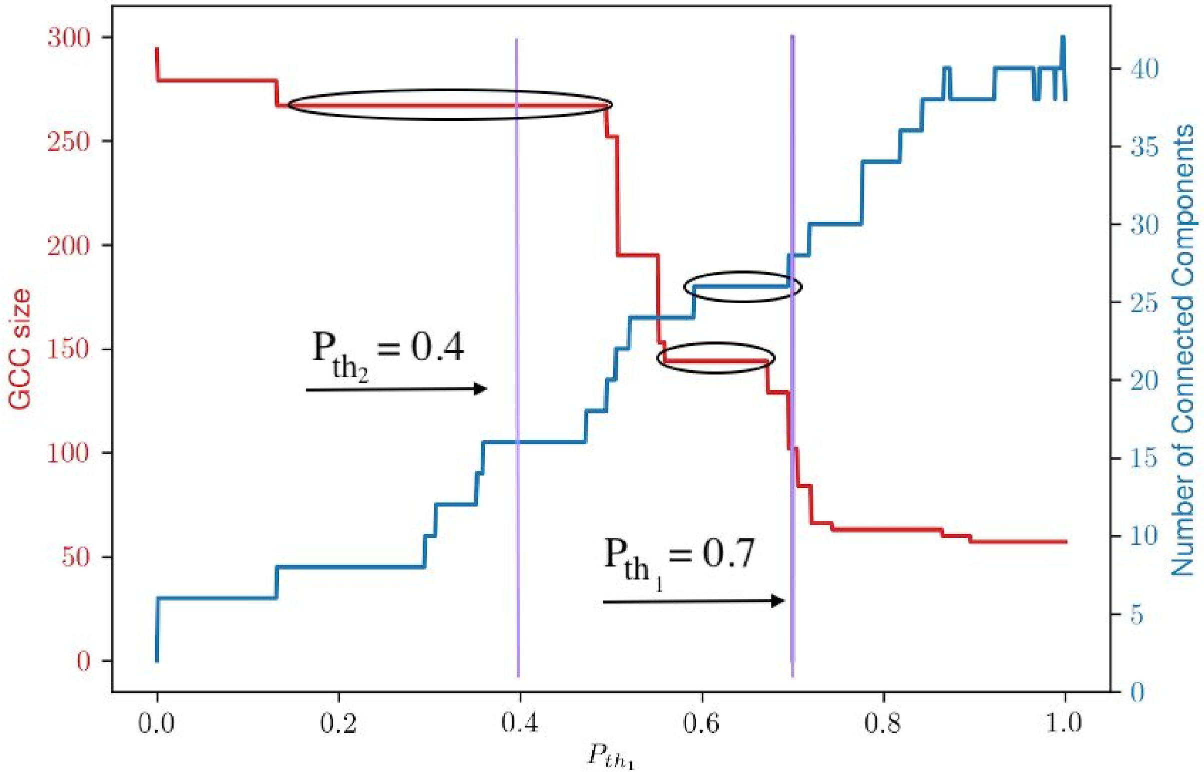

To determine a good threshold to get the core nodes, i,e. determining P_{th_1} , we need to meet two conditions: (i) The GCC size should not increase as the threshold is decreased from P_{th_1} ; in other words the GCC size has a long flat region to the left of P_{th_1} , and (ii) The number of connected components does not change as well, and the plot should have a corresponding flat region. As shown in the above figure this is satisfied if we set P_{th_1} = 0.7. The oval highlights point out the corresponding flat regions. Each connected component defines a set of core nodes. Figs. 10 and 8 in the main paper correspond to core nodes determined by P_{th_1} = 0.7.

In order to determine overlapping nodes, we consider a value of P_{th} where the GCC size has increased sharply, indicating that a lot of the sparsely connected nodes are being absorbed by the core communities. We pick P_{th_2} = 0.4 where the GCC size has a flat region.

Fig. 10 in the main paper corresponds to these choices of P_{th_1} and P_{th_2} .

For bridgegate, we again picked T_{max} = 1000 and the corresponding plots of GCC size and the number of connected components are shown below. As one can see, the GCC size has a sharp transition over a small region: $0.65 < P_{th} < 0.72$ where it goes from being a small structure to absorbing pretty much all the nodes. This is an indication of a so-called phase transition, where a small shift in threshold leads to a huge change in connectivity.

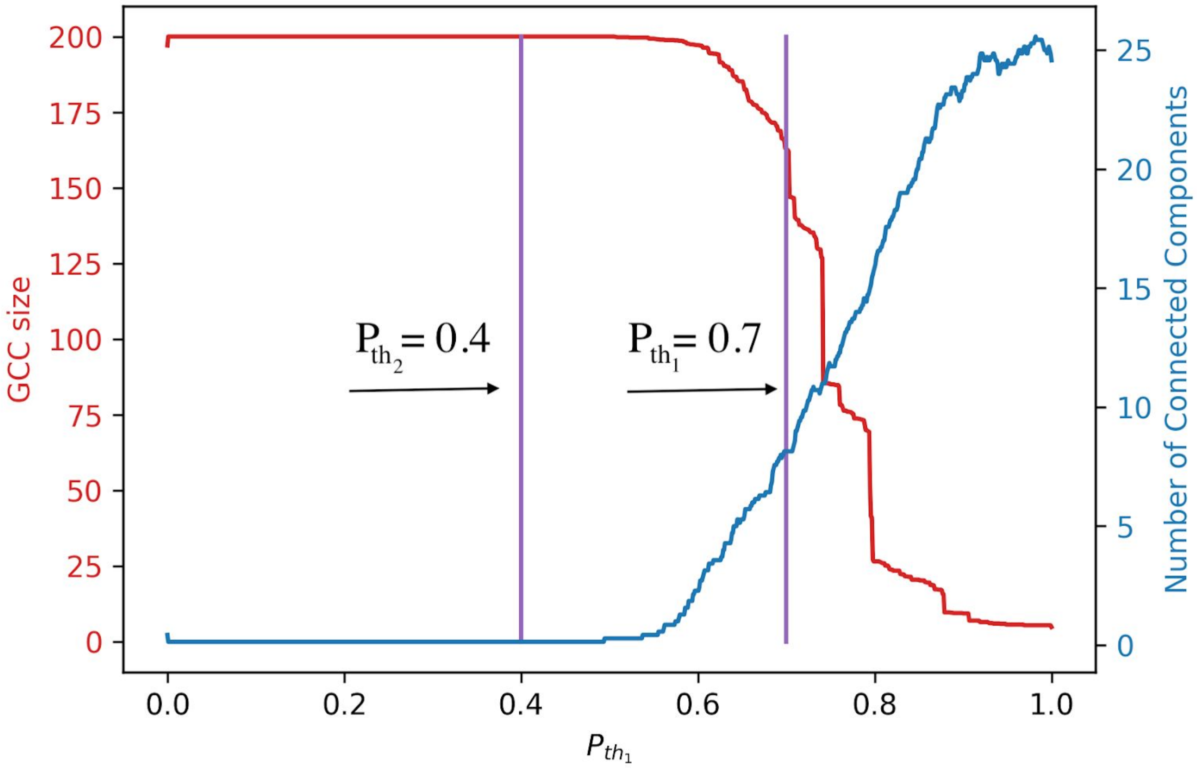

If one picks the same thresholds as in Pizzagate, then as shown in the following figure, the resulting community structure is dominated by one large community and everything gets connected via the overlapping nodes.

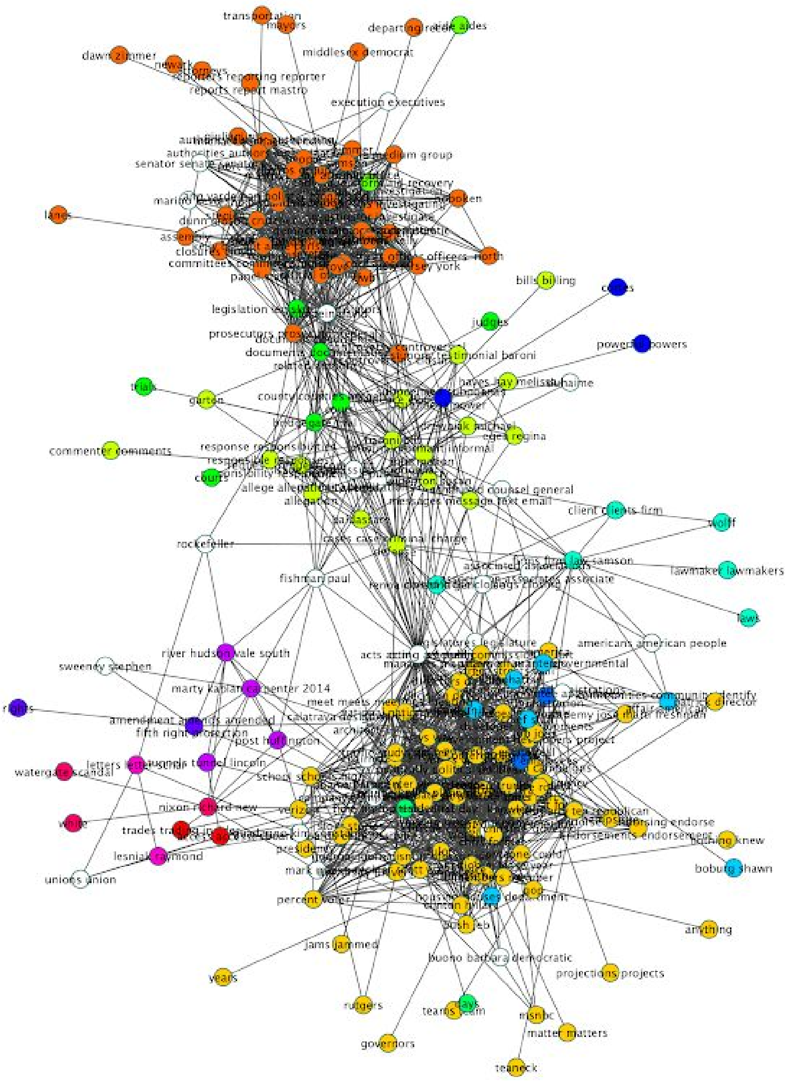

transportation
mayors
departing
aides
middlesex democrat
dawn zimmer
newark
reporters reporting reporter
reports report mastro
execution executives
senator senate
lanes
hoboken
officers
north
york
bills billing
legislation
prosecutors
judges
documents
timonial baroni
trials
garten
county counties
hayes
melissa
power
powers
commenter comments
response responsibility
courts
allege
allegation
messages message text email
counsel general
client clients firm
wolff
cases
criminal charge
rockefeller
fishman paul
firms
samson
lawmaker lawmakers
associate associates
laws
legislators legislature
americans american people
river hudson vale south
acts acting
sweeney stephen
marty kaplan center 2014
governmental
rights
amendment amends amended
fifth right protection
meet meets
director
post huffington
letters
lincoln
watergate scandal
nixon richard new
white
trades trading
lesniak raymond
unions union
verizon
presidency
mark
percent voter
endorsing endorse
endorsements endorsement
nothing knew
republican
bobulu shawn
department
bush jeb
anything
jams jammed
buono barbara democratic
years
projections projects
rutgers
teams team
msnbc
matter matters
governors
teaneck
